# On the Pathology of Acute Periostitis

**Published:** 1881-10

**Authors:** 


### ON THE PATHOLOGY OF ACUTE PERIOSTITIS.

Mr. C. T. Dent read a paper on this subject before the Surgical Society of London. He said that the exact relation which acute periostitis and osteo-myelitis bear to each other, as well as to the articular affections with which they are so often associated, have long been matters of dispute; so, also, have their effects with regard to the extent of necrosis which usually follows. French and German writers hold, generally, that total necrosis of the shaft will not take place unless osteo-myelitis has been present, as well as periostitis. This view is not shared for the most part by writers in this country, and cases are on record where the removal of the entire diaphysis has been performed for the results of acute periostitis alone. The exact tissue where the changes commence in acute periostitis is also a subject of difference of opinion; some, German writers especially, hold that it commences in the outer cellular layer of the periosteum, and destroys the fibrous layer by suppuration. This is, however, but one form of the disease frequently seen about the lower third of the femur, and also in true whitlow (panaritium periostale), More commonly it begins in the deeper osteogenetic layer and raises up the periosteum from the bone. This may lead to osteo-myelitis, and is sometimes, though not commonly, associated with affections of the neighboring joints. Acute idiopathic osteo-myelitis, as a primary affection, is rare in this country. In examining microscopically the periosteum in a case of acute periostitis unaccompanied by osteo-myelitis, the author found that the inflammatory changes were chiefly situated in the deeper parts; the fibrous layer was intact, though infiltrated, and new bone was developing in it. There was no active inflammation of bone or medulla; the changes observed in the latter, the author considered to be due to disintegration and breaking down of the tissues, owing to the cutting off of the vascular supply. The leg in this case had been amputated on the nineteenth day. Other specimens showed that the inflammation spreads from the outer to the deeper parts, chiefly around the smaller vessels. The author remarks that usually, no doubt, where these cases are met with, it is not uncommon to find periostitis, ostitis, and osteo-myelitis all existing together, yet it is none the less important to distinguish them from each other, as far as possible, and also from the form of disease attacking the outer cellular layer of the periosteum, for when modified by early treatment, the ultimate results are very different.—*Medical and Surgical Reporter.*

